# The effect of the COVID-19 pandemic on TNM status among head and neck cancer patients in Hungary

**DOI:** 10.3389/pore.2026.1612298

**Published:** 2026-02-20

**Authors:** Benedek Besenczi, Angéla Horváth, Imre Uri, Kornél Dános

**Affiliations:** Department of Otorhinolaryngology, Head and Neck Surgery, Semmelweis University, Budapest, Hungary

**Keywords:** COVID-19, head and neck cancers, oncologic pathways, TNM status, viral pandemics

## Abstract

**Purpose:**

Hungary ranks among the countries with both the highest incidence and mortality of head and neck cancers worldwide. The COVID-19 pandemic, caused by the SARS-CoV-2 virus placed a significant burden on the healthcare system. Our study aims to investigate its impact on Hungarian head and neck cancer patients by analyzing changes in stage at presentation, patient delay and overall survival due to the viral pandemic.

**Methods:**

A retrospective cohort study was performed analyzing patients’ medical records from a tertiary head and neck surgical center in Hungary. The inclusion criteria required the tumor to be a squamous cell carcinoma of the oral cavity, oropharynx, hypopharynx, or larynx. Based on the timing of restrictive measures due to the pandemic, patients were divided into two groups: Group A: “pre-COVID-19” (3 September 2012 – 11 March 2020) and Group B: “post-COVID-19 onset” (12 March 2020 – 5 December 2022) The latter group was further subdivided into Group C: “during-COVID-19” (12 March 2020 – 13 June 2021) and Group D: “post-COVID-19” (14 June 2021 – 5 December 2022).

**Results:**

620 patients met the inclusion criteria. Group A had 427 patients, Group B had 193, Group C had 69, and Group D had 124. Compared to Group A (54.1%), there was a higher proportion of N+ status patients in Group B (69.6%), Group C (63.8%), and Group D (73.0%), with a significant difference throughout. Changes in T status and patient delay time was not present. Analyzing symptoms, there was a significant increase in delay time for patients with hemoptysis (from 2.1 to 16.3 weeks). No significant difference in overall survival was observed between the study groups.

**Conclusion:**

There are limited publications available on this topic in Europe, particularly in Hungary, especially studies that compare the periods before, during, and after the COVID-19 pandemic. Head and neck cancer patients were found to have more advanced clinical nodal disease after the COVID-19 onset, despite no changes in patient delay time and overall survival. Our findings highlight the importance of further studies on how viral infections and pandemics affect oncology care pathways to improve preparedness for future public health crises.

## Introduction

Head and neck cancers are the 7th most common type of cancer worldwide, accounting for 4.6% of annual cancer-related mortality [1]. Hungary ranks third globally and first in Europe in the combined incidence of oral cavity, laryngeal, oropharyngeal, and hypopharyngeal cancers [2]. In terms of mortality from these cancers, Hungary ranks sixth globally and fourth in Europe [3].

A specific cancer type among head and neck cancers is human papillomavirus (HPV)-associated oropharyngeal cancer, whose incidence has been increasing since the 2000s. The typical locations are the tonsils and the base of the tongue, as the basal cell layer in these areas is easily accessible to HPV due to the invaginations present. A hallmark of these tumors is that they lack an *in-situ* phase and frequently metastasize to cystic lymph nodes, even in early T status. The overall survival of HPV-positive oropharyngeal cancers is better than that of HPV-negative cases. This is partially attributed to the fact that HPV-positive patients generally have a better socioeconomic status, consume less alcohol and tobacco, and are younger. Furthermore, these tumors are more sensitive to radiotherapy, chemotherapy, and immune-checkpoint inhibitory therapy [4–7].

The Coronavirus Disease 2019 (COVID-19) pandemic had a significant impact on the healthcare system. According to a World Health Organization (WHO) survey, the pandemic severely affected the care of non-coronavirus patients as well. This was further exacerbated by the suspension of preventive services. The accessibility of healthcare decreased, and disruptions in the treatment of certain diseases also emerged [8]. The pandemic placed a substantial burden on healthcare systems, and in many countries, government measures led to the reallocation of healthcare personnel to focus on pandemic management. Due to hospital overcrowding and fear of the virus, elective surgeries and treatments were postponed [9].

Hungary reported its first confirmed case of coronavirus infection on March 4, 2020, followed by the declaration of a nationwide state of emergency on March 11, 2020. A series of healthcare-related restrictions were subsequently introduced, including a complete prohibition of hospital visitations, the designation of specific hospitals exclusively for the treatment of COVID-19 patients, the suspension of all elective medical procedures, the withdrawal of healthcare workers aged over 65 years from direct patient care activities, and the mandatory release of 50%–60% of hospital bed capacity to accommodate COVID-19 cases. The final healthcare-related restriction was lifted on June 13, 2021 [10].

The Tumor-Node-Metastasis (TNM) stage and patient delay time (the interval from symptoms onset to the first medical consultation) are important prognostic factors for head and neck cancers [11–13]. Numerous studies have highlighted that these factors may have changed in relation to the COVID-19 pandemic [14–21].

The indirect effects of the pandemic are still not fully understood. We hypothesize that head and neck cancer patients presented later to the healthcare system. Therefore, we expected changes in tumor stage, patient delay, and survival. The aim of our current study was to examine the initial TNM status and patient delay of head and neck cancer patients and to assess any changes due to the impact of COVID-19 at a tertiary head and neck surgical referral center in Hungary.

## Methods

In this retrospective cohort study, all patients were enrolled who had histological diagnosis of head and neck cancer (oral cavity, oropharynx, hypopharynx or larynx) between 3 September 2012, and 5 December 2022, at the Department of Otorhinolaryngology, Head and Neck Surgery, Semmelweis University, Budapest, Hungary. Included patients had to be diagnosed at our department, and patients with a history of prior head and neck malignancies or previous oncological treatments were excluded. Patients were selected retrospectively, and the database included all patients who appeared at our multidisciplinary tumor board between 2012 and 2022 with the cancer of the oral cavity, oropharynx, larynx and hypopharynx.

The patient group under investigation was divided into two periods: the first is the “pre-COVID-19” period (Group A), spanning from September 3, 2012 to March 11, 2020. The start of the COVID-19 period was defined as March 11, 2020, as this was the date when the Hungarian government declared a nationwide state of emergency. The second patient group corresponds to the “post-COVID-19 onset” period (Group B), which extends from March 12, 2020 to December 5, 2022. To examine changes in certain parameters, the post-COVID-19 onset group was further divided into two subperiods: the “during COVID-19” period (Group C), from 12 March 2020 to 13 June 2021, and the “post-COVID-19” period (Group D), from 14 June 2021 to 5 December 2022. 13 June 2021 was marked as the end date of the COVID-19 pandemic, as this was the day all health-related restrictions were lifted.

Patients’ medical records (outpatient notes, discharge summaries, multidisciplinary tumor board reports, histological and imaging reports) were evaluated. Mortality data were retrieved from the Hungarian Cancer Registry. Data on the patients' age, gender, smoking and alcohol consumption habits, tumor region, TNM status, tumor size, symptoms, the onset date of their complaints, the date of their first outpatient visit and, if applicable, the date of death were collected. De-identified data stored securely. The interval calculated from the onset of symptoms to the first outpatient visit was defined as the patient delay time.

To overcome the difficulty arising from the shift from the 7th to the 8th TNM edition, tumor staging was simplified to early stage (T1–2) and locally advanced (T3–4) categories. Nodal involvement was recorded as absent (N0) or present (N+). Metastatic disease was defined as negative (M0) or positive (M1) [22].

To determine the HPV status in oropharyngeal cancers, as in the clinical practice and current guidelines, immunohistochemical detection of the tumor suppressor p16 was applied (at least 70% combined nuclear and cytoplasmic staining of the tumor tissue was necessary for positivity). This method is a reliable, surrogate marker for testing human papillomavirus (HPV) in oropharyngeal carcinoma [6].

Statistical analysis of the data was conducted using IBM SPSS Statistics for macOS (IBM Corp., Armonk, NY, version 29). Descriptive statistics, along with analysis of symptom-related variables and tumor characteristics (TNM, region), were performed using Chi-square tests (χ^2^). Data normality was assessed using the Shapiro–Wilk test. Age, which followed a normal distribution, was analyzed using an independent samples t-test. As most other parameters did not follow normal distribution, patient delay times and tumor sizes were analyzed with the non-parametric Mann–Whitney U test and the Kruskal–Wallis test. For survival analyses, overall survival data were used, and Kaplan-Meier estimates and Log-rank tests were calculated. The cutoff date for overall survival analysis was 3rd July 2025. A univariate Cox regression model was applied to evaluate the impact of the studied risk factors, with the statistical event defined as death (including cancer-related and non-cancer-related deaths). For continuous variables, the median, mean, and range were reported, whereas for categorical variables, absolute and percentage frequencies were presented. A 95% confidence interval (CI) was applied. A *p* < 0.05 value was considered statistically significant.

This research was carried out with the ethical permission of Semmelweis University (SE IKEB 105/2014).

## Results

### Patient characteristics

**FIGURE 1:**
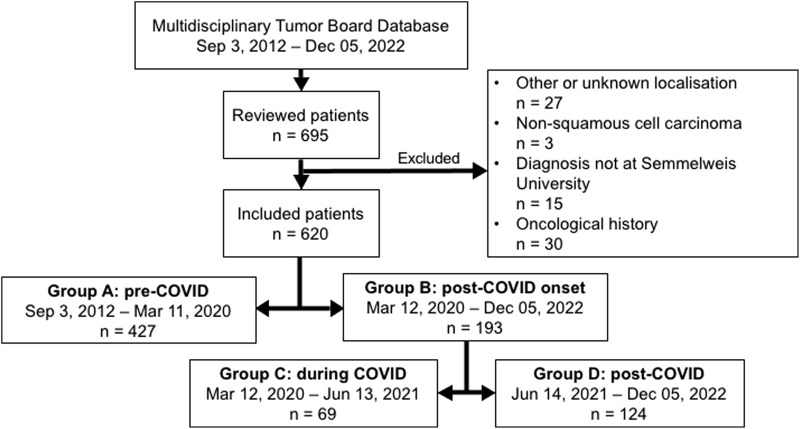
Flow chart demonstrating the population cohort.

A total of 695 patients were identified for enrollment into this study. Of these, 15 were excluded due to having their histopathological diagnosis done at an external clinic. Additionally, 9 patients had a prior history of head and neck malignancy, 19 had previously undergone oncological treatment, and 2 had both a history of head and neck malignancy and prior oncological treatment. Moreover 21 individuals were excluded due to a cancer of unknown primary (CUP), 6 individuals due to other head and neck tumor localizations (e.g., nasopharynx), and 3 individuals because their tumor was not a squamous cell carcinoma. This resulted in a final sample size of 620 patients. Overall, there are 427 patients in Group A, 193 patients in Group B, 69 patients in Group C, and 124 patients in Group D. The derivation of our final study cohort is demonstrated in Figure 1.

Age was normally distributed (*p* = 0.204), while tumor size (*p* < 0.001) and patient delay time (*p* < 0.001) significantly deviated from normality according to the Shapiro–Wilk test.

For a detailed overview of patient characteristics, refer to Table 1. An independent samples t-test was performed for patients’ age, and Chi-square tests were used for gender distribution, smoking and alcohol consumption habits, tumor localization, presenting symptoms and p16 status. Among the enrolled patients, 80.3% were male and 19.7% were female, with a median age of 63 years. Based on smoking habits, the proportion of never smokers increased from 6.7% to 17.1%, former smokers changed from 18.7% to 20.0%, while the proportion of current smokers decreased from 74.6% to 62.9% in Group B compared with Group A (*p* < 0.001). Regarding alcohol consumption habits, there was no difference between the two groups (*p* = 0.233). A total of 40.0% of patients had never consumed alcohol regularly, 10.6% had done so in the past, and 49.4% were current regular consumers.

**TABLE 1:** Demographic and clinical characteristics of patients involved in our study.

​	Total	Group A	Group B	*p*
Total number	620	427	193	​
Sex	​	​	​	0.273
Male	498 (80.3%)	348 (81.5%)	150 (77.7%)	​
Female	122 (19.7%)	79 (18.5%)	43 (22.3%)	​
Age	​	​	​	0.120
Median (range)	63 years (41–88)	62 years (41–88)	64 years (42–83)	​
Smoking	​	​	​	<0.001
Never	57 (9.8%)	27 (6.7%)	30 (17.1%)	​
Previously	111 (19.1%)	76 (18.7%)	35 (20.0%)	​
Currently	413 (71.1%)	303 (74.6%)	110 (62.9%)	​
Not available	39	21	18	​
Alcohol consumption	​	​	​	0.233
Never	218 (40.0%)	141 (37.6%)	77 (45.3%)	​
Previously	58 (10.6%)	42 (11.2%)	16 (9.4%)	​
Currently	269 (49.4%)	192 (51.2%)	77 (45.3%)	​
Not available	75	52	23	​
Region	​	​	​	0.034
Oral cavity	58 (9.4%)	40 (9.4%)	18 (9.3%)	​
Oropharynx	234 (37.7%)	146 (34.2%)	88 (45.6%)	​
Hypopharynx	110 (17.7%)	84 (19.7%)	26 (13.5%)	​
Supraglottic larynx	64 (10.3%)	46 (10.8%)	18 (9.3%)	​
Glottic larynx	141 (22.7%)	98 (23.0%)	43 (22.3%)	​
Transglottic larynx	8 (1.3%)	8 (1.9%)	0 (0.0%)	​
Subglottic larynx	5 (0.8%)	5 (1.2%)	0 (0.0%)	​
Symptoms				
Sore throat	142 (22.9%)	99 (23.2%)	43 (22.3%)	0.804
Hoarseness	190 (30.6%)	132 (30.9%)	58 (30.1%)	0.829
Neck mass	192 (31.0%)	112 (26.2%)	80 (41.5%)	<0.001
Difficulty in swallowing	178 (28.7%)	122 (28.6%)	56 (29.0%)	0.910
Weight loss	56 (9.0%)	24 (5.6%)	32 (16.6%)	<0.001
Hemoptysis	18 (2.9%)	9 (2.1%)	9 (4.7%)	0.079
Earache	60 (9.7%)	40 (9.4%)	20 (10.4%)	0.698
Trismus	4 (0.6%)	2 (0.5%)	2 (1.0%)	0.413
Dyspnea	41 (6.6%)	30 (7.0%)	11 (5.7%)	0.538
p16 status	​	​	​	0.009
Negative	205 (71.2%)	146 (76.4%)	59 (60.8%)	​
Positive	83 (28.8%)	45 (23.6%)	38 (39.2%)	​
Not available	332	236	96	​

Independent samples t-test was performed for age, and Chi-square tests were performed for all other variables. (Group A: pre-COVID-19; Group B: post-COVID-19, onset).

In terms of tumor sites, the most common within the cohort was the oropharyngeal region, the proportion of which increased from 34.2% to 45.6% in Group B compared with Group A. The proportion of hypopharyngeal tumors showed a decreasing trend, from 19.7% to 13.5%. No notable trend was observed in the other regions: oral cavity tumors accounted for 9.4% of all enrolled cases, supraglottic laryngeal tumors for 10.3%, glottic tumors for 22.7%, transglottic tumors for 1.3%, and subglottic tumors for 0.8%.

Regarding presenting symptoms, among all patients, 22.9% reported sore throat, 30.6% hoarseness, 31.0% neck mass, 28.7% difficulty in swallowing, 9.0% weight loss, 2.9% hemoptysis, 9.7% earache, 0.6% trismus, and 6.6% dyspnea. A significant difference between the two groups was observed only in the increased proportion of patients presenting with neck mass (Group A: 26.2%; Group B: 41.5%) and weight loss (Group A: 5.6%; Group B: 16.6%) in Group B compared to Group A. Additionally, a non-significant increasing trend was noted in the case of hemoptysis (Group A: 2.1%; Group B: 4.7%).

In terms of p16 status, 23.6% of tumors in group A were positive, while in group B, this significantly rose to 39.2% (p = 0.009).

### The associations between cancer stage, tumor size and study groups

Examining tumor TNM stages by Chi-square tests, there was not a significant (*p* = 0.293) difference in the T3-4 status patients between Groups A (57.2%) and B (61.8%). However, a significant difference (*p* < 0.001) was observed in the initial N status between the two groups. In Group A, 45.9% of cases were N0, while 54.1% were N+. In Group B, 30.4% were N0 and 69.6% were N+.

Despite not finding a significant change in T status, tumor size (greatest dimension) showed a significant (*p* = 0.004) difference according to the Mann-Whitney U test (Group A median was 28 mm, Group B median was 35 mm). The M status did not show significant difference (*p* = 0.590). See details in Table 2.

**TABLE 2:** TNM stage distribution and tumor size in Group A and B.

​	Total	Group A	Group B	*p*
T	​	​	​	0.293
T1-2	250 (41.3%)	177 (42.8%)	73 (38.2%)	​
T3-4	355 (58.7%)	237 (57.2%)	118 (61.8%)	​
Not available	15	13	2	​
N	​	​	​	<0.001
N0	249 (41.0%)	191 (45.9%)	58 (30.4%)	​
N+	358 (59.0%)	225 (54.1%)	133 (69.6%)	​
Not available	13	11	2	​
M	​	​	​	0.590
M0	566 (93.4%)	391 (93.8%)	175 (92.6%)	​
M1	40 (6.6%)	26 (6.2%)	14 (7.4%)	​
Not available	14	10	4	​
Size	​	​	​	0.004
Median (range)	30 mm (2–140)	28 mm (2–140)	35 mm (2–90)	​

Mann-Whitney U test was performed for size, and Chi-square tests were performed for all other variables. (Group A: pre-COVID-19; Group B: post-COVID-19, onset).

For more detailed analyses, Group B (post-COVID-19 onset) was divided into Group C (during COVID-19) and Group D (post-COVID-19) subgroups. Performing the Chi-square tests in the three-group analysis, the change in T status remained non-significant (*p* = 0.442), while the change in N status remained significant (*p* < 0.001). In Group C, 36.2% of cases were N0, and 63.8% were N+, whereas in Group D, 27.0% were N0 and 73.0% were N+ (Table 3).

**TABLE 3:** T and N stage distribution in Group A, C and D.

​	Total	Group A	Group C	Group D	*p*
T	​	​	​	​	0.442
T1-2	250 (41.3%)	177 (42.8%)	24 (34.8%)	49 (40.2%)	​
T3-4	355 (58.7%)	237 (57.2%)	45 (65.2%)	73 (59.8%)	​
Not available	15	13	0	2	​
N	​	​	​	​	<0.001
N0	249 (41.0%)	191 (45.9%)	25 (36.2%)	33 (27.0%)	​
N+	358 (59.0%)	225 (54.1%)	44 (63.8%)	89 (73.0%)	​
Not available	13	11	0	2	​

Chi-square tests were performed. (Group A: pre-COVID-19; Group C: during COVID-19; Group D: post-COVID-19).

Changes in N and T categories broken down by tumor regions were also investigated by Chi-square tests. In T category a trend could be observed for supraglottic laryngeal tumors (T3-4 Group A: 53.3%; Group B: 77.8%; *p* = 0.073), but there were no significant changes in all other regions. For oral cavity (*p* = 0.031), oropharyngeal (*p* = 0.013), and glottic laryngeal tumors (*p* = 0.009), a significant increase in the N+ category was observed in Group B compared to Group A. No significant difference was observed in the other tumor regions. For detailed data, see Table 4.

**TABLE 4:** T and N stage distribution in Group A and B by tumor regions.

​	Total	Group A	Group B	*p*
Oral cavity				
T	​	​	​	0.579
T1-2	22 (38.6%)	16 (41.0%)	6 (33.3%)	​
T3-4	35 (61.4%)	23 (59.0%)	12 (66.7%)	​
Not available	1	1	0	​
N	​	​	​	0.031
N0	25 (43.1%)	21 (52.5%)	4 (22.2%)	​
N+	33 (56.9%)	19 (47.5%)	14 (77.8%)	​
Oropharynx				
T	​	​	​	0.455
T1-2	96 (42.7%)	62 (44.6%)	34 (39.5%)	​
T3-4	129 (57.3%)	77 (55.4%)	52 (60.5%)	​
Not available	9	7	2	​
N	​	​	​	0.013
N0	42 (18.7%)	33 (23.7%)	9 (10.5%)	​
N+	183 (81.3%)	106 (76.3%)	77 (89.5%)	​
Not available	9	7	2	​
Hypopharynx				
T	​	​	​	0.508
T1-2	24 (22.2%)	17 (20.7%)	7 (26.9%)	​
T3-4	84 (77.8%)	65 (79.3%)	19 (73.1%)	​
Not available	2	2	0	​
N	​	​	​	0.582
N0	29 (26.6%)	21 (25.3%)	8 (30.8%)	​
N+	80 (73.4%)	62 (74.7%)	18 (69.2%)	​
Not available	1	1	0	​
Supraglottic larynx				
T	​	​	​	0.073
T1-2	25 (39.7%)	21 (46.7%)	4 (22.2%)	​
T3-4	38 (60.3%)	24 (53.3%)	14 (77.8%)	​
Not available	1	1	0	​
N	​	​	​	0.418
N0	26 (41.3%)	20 (44.4%)	6 (33.3%)	​
N+	37 (58.7%)	25 (55.6%)	12 (66.7%)	​
Not available	1	1	0	​
Glottic larynx				
T	​	​	​	0.431
T1-2	78 (56.1%)	56 (58.3%)	22 (51.2%)	​
T3-4	61 (43.9%)	40 (41.7%)	21 (48.8%)	​
Not available	2	2	0	​
N	​	​	​	0.009
N0	117 (84.2%)	86 (89.6%)	31 (72.1%)	​
N+	22 (15.8%)	10 (10.4%)	12 (27.9%)	​
Not available	2	2	0	​

Chi-square tests were conducted. (Group A: pre-COVID-19; Group B: post-COVID-19, onset).

### Comparison of patient delay during the study periods

Analyzing the median patient delay times, no significant difference (*p* = 0.604) was found between Groups A and B in the two-group comparison using the Mann-Whitney U test. Similarly, no significant changes (*p* = 0.793) were observed among Groups A, C, and D in the three-group comparison by Kruskal-Wallis test. An overview of the exact values is shown in Table 5.

**TABLE 5:** Patient delay (weeks) mean, median and range in the two-groups and three-groups analysis.

​	Total	Group A	Group B	Group C	Group D	*p*
Two-groups patient delay (weeks)	​	​	​	​	​	0.604
Mean	16.52	15.91	17.88	-	-	​
Median	9.07	8.86	9.71	-	-	​
Range	0–223.9	0–209.7	0–223.9	-	-	​
Three-groups patient delay (weeks)	​	​	​	​	​	0.793
Mean	16.52	15.91	-	18.59	17.49	​
Median	9.07	8.86	-	8.14	9.79	​
Range	0–223.9	0–209.7	-	0–223.9	0–122.1	​

Mann-Whitney U test and Kruskal-Wallis test were performed. (Group A: pre-COVID-19; Group B: post-COVID-19 onset; Group C: during COVID-19; Group D: post-COVID-19).

The median patient delay time was the longest for subglottic laryngeal tumors (31.4 weeks), followed by glottic laryngeal tumors (14 weeks). The shortest delay times were observed for oropharyngeal (7.6 weeks) and oral cavity tumors (7.7 weeks). There was no significant change in delay time for any localization between Groups A and B.

Regarding symptoms, the longest delay was observed in patients with hoarseness (13.8 weeks), followed by those with trismus (11 weeks). The shortest delays were reported by patients with dyspnea (7.4 weeks) and hemoptysis (7.4 weeks). The delay time for patients with hemoptysis (Group A: 2.1 weeks, Group B: 16.3 weeks) significantly (*p* = 0.009) increased between the two study periods.

For further distribution of delay times by symptoms and primary tumor localization in the study groups, see Table 6. In both cases Mann-Whitney U tests were performed.

**TABLE 6:** Median patient delay (weeks) according to tumor region and symptoms.

​	Total	Group A	Group B	*p*
Patient delay (weeks) median				
Oral cavity	7.7	7	9.9	0.470
Oropharynx	7.6	7.3	8.5	0.798
Hypopharynx	9.9	9.4	13	0.151
Supraglottic larynx	8.7	8.6	8.8	0.469
Glottic larynx	14	14.1	14	0.350
Transglottic larynx	8.1	8.1	-	-
Subglottic larynx	31.4	31.4	-	-
Patient delay (weeks) median				
Sore throat	9.7	9.6	9.7	0.418
Hoarseness	13.8	13.8	13.6	0.516
Neck mass	8.9	8.9	9	0.712
Difficulty in swallowing	8.7	8.5	9.7	0.159
Weight loss	8.8	8.8	9.1	0.888
Hemoptysis	7.4	2.1	16.3	0.009
Earache	8.6	8.7	7.9	0.515
Trismus	11	32.6	7.2	0.439
Dyspnea	7.4	8.6	7	0.508

Mann-Whitney U tests were performed. (Group A: pre-COVID-19; Group B: post-COVID-19, onset).

### Survival analysis

**FIGURE 2:**
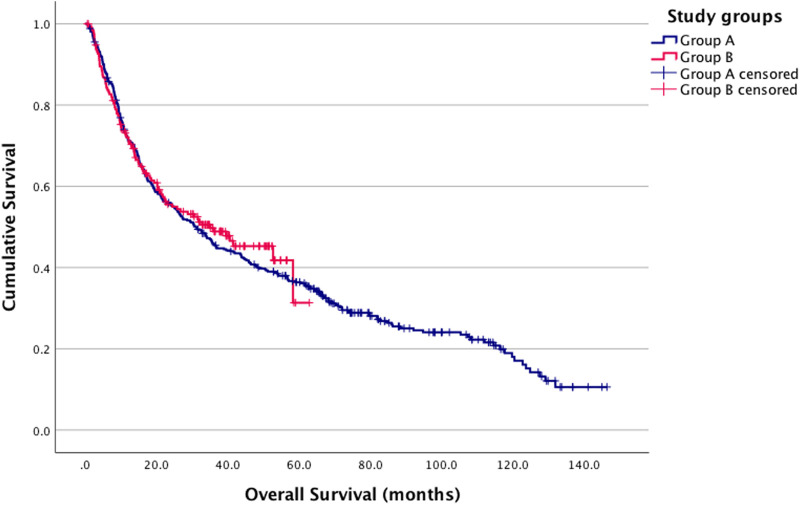
Kaplan-Meier estimates. Group A and B survival analysis log-rank *p* = 0.664 (Group A (blue): pre-COVID-19; Group B (red): post-COVID-19 onset).

**FIGURE 3:**
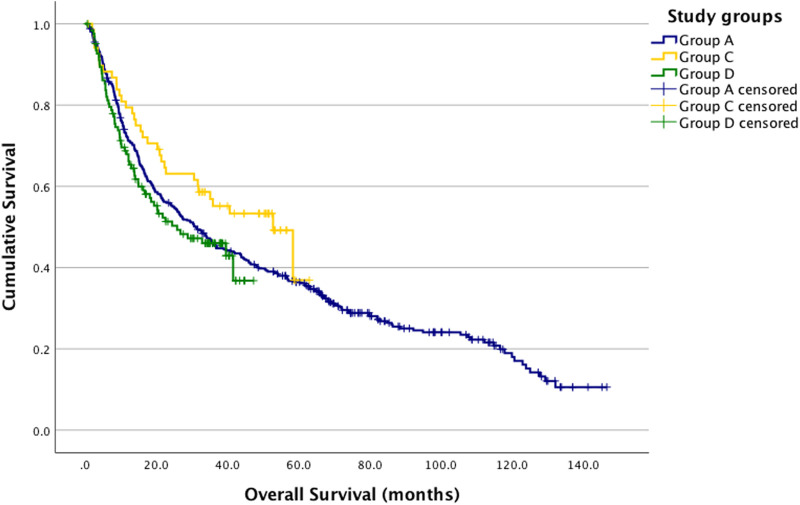
Kaplan-Meier estimates. Group A, C and D survival analysis log-rank *p* = 0.168. A vs. C *p* = 0.106; A vs. D *p* = 0.464; C vs. D *p* = 0.5067 (Group A (blue): pre-COVID-19; Group C (yellow): during COVID-19; Group D (green): post-COVID-19).

When comparing overall survival between Groups A and B using the log-rank test, no significant difference was observed (p = 0.664) (Figure 2). Similarly, no significant difference was found in the three-group analysis (p = 0.168) (Figure 3).

In the univariate Cox regression analysis, the survival of other patient groups was compared to Group A. The hazard ratio (HR) for Group B was 0.949, Group C had an HR of 0.744, for Group D, the HR was 1.110. None of these results were statistically significant (Table 7).

**TABLE 7:** Univariate Cox regression analysis for overall survival.

​	*p*	Hazard ratio (HR)	95% confidence interval (CI)	
			Lower	Upper
Group A	​	1 (reference)	​	​
Group B	0.664	0.949	0.751	1.201
Group C	0.110	0.744	0.518	1.070
Group D	0.458	1.110	0.842	1.465

Group A: pre-COVID-19; Group B: post-COVID-19 onset; Group C: during COVID-19; Group D: post-COVID-19.

## Discussion

This study illustrates the impact of COVID-19 on the TNM status of patients with head and neck cancer. Our key finding is the shift toward higher nodal status following the onset of COVID-19. Examining the results in more detail, we found that the N+ status increased compared to the pre-COVID-19 period during the COVID-19 era and continued to rise after COVID-19. This result is consistent with the observed increase in the proportion of patients presenting with a neck mass. Regarding T status there was no significant difference between the study groups. Also, we experienced a shift in the proportion of tumor sites of origin: the number of oropharyngeal cancers diagnosed increased markedly. Nevertheless these tumors lack an *in-situ* phase and tend to present with lymph node metastases even when the primary lesion is small [5]. To rule out the confounding effect of rising oropharyngeal cancer number on the increasing N+ stage cancers, we assessed the changes in nodal status by tumor sites: the rise in the number of N+ cases remained significant in oral cavity, oropharyngeal and glottic laryngeal cancers. Data consistent with our findings have been reported by Stevens et al. [16], and several studies also describe changes in T status alongside N status [14, 15]. Certain articles report only changes in T status [17, 23], while multiple papers found no change in the presentation stage of head and neck cancers relative to COVID-19 [19, 20, 24, 25]. These discrepancies across studies may be explained by differences in healthcare organization, patient pathways, and screening accessibility during the pandemic, as well as by varying lockdown measures, time periods analyzed, and sample sizes. Moreover, the heterogeneity of tumor sites and stages included in the studies, along with regional differences in public health responses and diagnostic capacities, may also contribute to the inconsistent findings.

Comparing the median delay times, the difference was not significant between the study groups. In contrast, several other studies reported significant changes in patient delay times [15, 18, 21]. At first glance, the increase in N+ cases despite unchanged delay times and survival may appear contradictory. However, this could be explained by changes in diagnostic and referral pathways rather than patient behavior. Although patients sought care within a similar timeframe, restricted access to specialized diagnostics may have led to more advanced nodal stages at presentation. Moreover, the rising proportion of oropharyngeal cancers–tumors prone to early nodal metastasis–may have contributed to the higher N+ rates [26, 27].

Patient delay times by site of origin did not change significantly due to COVID-19; however, the longest delays were observed in subglottic and glottic laryngeal tumors, while the shortest were seen in oral cavity and oropharyngeal sites. This may be because laryngeal malignancies often cause hoarseness, which is not as alarming, in contrast to symptoms more commonly associated with oral cavity and oropharyngeal tumors, such as neck masses or sore throat, which tend to raise more concern among patients. Moreover, glottic tumors can harbour a less aggressive growth pattern compared to other localizations [28].

When examining patient delay time by symptoms in response to the pandemic, we observed a significant increase in delay for those with hemoptysis. However, the total number of hemoptysis cases was low, and therefore no definitive conclusions can be drawn from this observation. The overall shortest delays were observed among patients with symptoms like dyspnea and hemoptysis, likely because these are alarming symptoms. The longest delays were associated with hoarseness and trismus, which are symptoms less likely to raise immediate concern and are often attributed to smoking by patients. The prolonged delay correlates with the longer delay times observed in laryngeal tumors. This underscores the importance of early recognition and awareness of early warning symptoms, which can significantly influence the timely diagnosis and treatment of head and neck cancers. Educating patients and healthcare professionals about these key warning signs is crucial for reducing diagnostic delays and improving outcomes.

Analyzing survival, no significant change was found in survival rates in the COVID-19 group compared to other groups. This aligns with the fact that cancer care in our clinic remained largely unaffected by the direct impact of COVID-19 restrictions, thanks to the continued efforts to provide essential oncology services. Despite the challenges posed by the pandemic, the mainly uninterrupted treatment of cancer patients may have played a crucial role in maintaining survival outcomes during this period. Additionally, due to the suspension of elective procedures, patients were able to receive earlier surgical appointments, potentially leading to more timely interventions and better outcomes for certain cases. A Brazil study reported an increase in mortality rates in 2020 compared to 2019, potentially attributed to the effects of COVID-19 [17]. However, several other studies found no difference in survival [29, 30].

Our study lays the groundwork for understanding the effects of the COVID-19 pandemic on head and neck cancer patients and underscores the need for enhanced preparedness for future viral pandemics and healthcare disruptions. We recommend the development of a comprehensive action plan that includes a prioritization protocol for oncology patients to safeguard their access to care, the implementation of a telemedicine-based triage system, and the launch of public awareness campaigns to educate patients about red-flag symptoms requiring urgent evaluation — particularly because early symptoms of head and neck cancers can easily be mistaken for signs of common upper respiratory infections.

A possible limitation of our study is its retrospective nature, and the fact that the onset of symptoms was reported subjectively by the patients, which may introduce recall bias. Additionally, patients from the COVID-19 onset period have a significantly shorter follow-up time compared to earlier cases. However, the study site is a tertiary cancer care provider, receiving patients from all parts of the country, thus representing the entire Hungarian population.

## Conclusion

To our knowledge, there are limited publications available about the impact of COVID-19 on TNM status of head and neck cancer patients in Central Europe, particularly in Hungary, especially those comparing the periods before, during, and after the COVID-19 pandemic. We found an increase in the proportion of patients with N+ status after the onset of COVID-19. Regarding tumor sites, the proportion of oropharyngeal cancers increased, while the occurrence of hypopharyngeal tumors decreased. In terms of presenting symptoms, the proportion of patients reporting neck mass and weight loss also increased. Overall, there was no difference in delay times; nevertheless, patients presenting with hemoptysis experienced increased delays, which was not documented before. Regarding survival, we found no significant change due to the pandemic. Consequently, it is of utmost importance to ensure the continuity of care for oncology patients during viral pandemics and healthcare crises. In the future, it would be advisable to include additional head and neck cancer centers from Hungary and other countries and to study survival with a longer follow-up period. Furthermore, future studies are needed to investigate the impact of viral infections and pandemics on the TNM status of patients with different types of cancer.

## Data Availability

The raw data supporting the conclusions of this article will be made available by the authors, without undue reservation.
